# Micronutrient Absorption and Related Outcomes in People with Inflammatory Bowel Disease: A Review

**DOI:** 10.3390/nu11061388

**Published:** 2019-06-20

**Authors:** Kyle Kilby, Holly Mathias, Lindsay Boisvenue, Courtney Heisler, Jennifer L. Jones

**Affiliations:** 1 Faculty of Medicine, Dalhousie University, 1459 Oxford Street, Halifax, NS B3J 4R2, Canada; Kyle.Kilby@dal.ca; 2 School of Health and Human Performance, Dalhousie University, 6230 South Street, Halifax, NS B3H 1T8, Canada; Holly.Mathias@dal.ca; 3 Seaway Valley Community Health Care, 353 Pitt Street, Cornwall, ON K6J 3R1, Canada; BoisvenueL@seawayvalleychc.ca; 4 Nova Scotia Collaborative Inflammatory Bowel Disease Program, Division of Digestive Care and Endoscopy, QEII Health Science Centre, Room 932, Victoria Building, 1276 South Park Street, Halifax, NS B3H 2Y9, Canada; courtney.heisler@nshealth.ca

**Keywords:** Inflammatory Bowel Disease, micronutrients, vitamin, mineral, deficiency

## Abstract

Inflammatory Bowel Disease (IBD) is a chronic disorder associated with immune dysregulation and chronic inflammation of the digestive tract. While it is poorly understood, the role of nutrition and nutrient status in the etiology of IBD and its associated outcomes has led to increased research relating to micronutrient deficiency. This review offers an overview of recent literature related to micronutrient absorption and outcomes in adults with IBD. Although the absorption and IBD-related outcomes of some micronutrients (e.g., vitamin D and iron) are well understood, other micronutrients (e.g., vitamin A) require further research. Increased research and clinician knowledge of the relationship between micronutrients and IBD may manifest in improved nutrient screening, monitoring, treatment, and outcomes for people living with IBD.

## 1. Introduction

Inflammatory Bowel Diseases (IBD) (including Crohn’s Disease (CD) and Ulcerative Colitis (UC)) are complex disorders involving immune dysregulation, leading to infiltration and destruction of the gastrointestinal tract. At its core, IBD results from an imbalance between pro-inflammatory and anti-inflammatory signaling, however the etiology of this imbalance, and of IBD itself, remains poorly understood [1]. An expanding body of literature implicates a shared role of genetics and the environment in the development of disease. The contributing role of environmental factors in the pathogenesis of IBD is further substantiated by observed rising IBD incidence rates in populations that have immigrated from geographic regions of low disease prevalence to regions where the disease is more prevalent [2]. Population-based studies highlight an association between increased intake of total fat, processed meat, and protein (common components of ‘Western’ diets), and the incidence of IBD [3,4].

In turn, IBD itself affects nutrition intrinsically by disrupting the absorption of nutrients in the gut, and extrinsically through reduced total food intake due to pain and fatigue, as well as the avoidance of food groups that exacerbate their symptoms [5,6,7,8,9,10,11]. Given differences in disease location between CD (commonly involving the ileocecal junction but can affect any part of the gastrointestinal tract) and UC (localized to the rectum and colon), and the distribution of nutrient absorption sites within the gut, the spectrum of the association between IBD and nutrient status varies in both magnitude and selectivity [12,13]. Micronutrient status is further impacted by increased gastrointestinal losses and the surgical treatment of IBD itself, which decreases the amount of mucosal surface area available for absorption [14]. The effect of selected micronutrient deficiencies on patient outcomes has been difficult to characterize. The severity of deficiency often moves lockstep with disease severity, making it difficult to draw conclusions about the specific impact of micronutrient levels on patient outcomes without experimental interventional studies to eliminate known or residual confounding effect. This review seeks to highlight many of the common micronutrient deficiencies associated with IBD and examine the reciprocal relationship between micronutrients and IBD with respect to absorption and outcomes. Furthermore, this review aims to examine current recommendations for micronutrient supplementation and screening for patients with IBD.

## 2. Literature Review

### 2.1. Materials & Methods

An initial literature search was performed in order to develop a set of well-studied and clinically relevant micronutrients. Medline, Pubmed, Embase and CINAHL databases were searched for English language peer-reviewed articles published during 2009–2019 using the keywords ‘inflammatory bowel disease’ and, ‘micronutrients’ alongside, ‘deficiency’, ‘outcomes’, ‘screening’, and/or ‘treatment’. This search strategy resulted in 1334 articles. Articles were excluded if they did not focus on the adult IBD patients or patient outcomes. From this, a subset of 160 papers were used to determine which micronutrient deficiencies warranted further investigation. 

Individual literature searches for each micronutrient were performed, with no exclusion criteria. Each search followed the same formula of keywords, with ‘inflammatory bowel disease’ and the vitamin in question, as well as the modifiers, ‘outcomes’, ‘deficiency’, ‘supplementation’, or ‘therapy’. From these subsearches, all relevant results were included. Finally, snowballing was used to find additional sources that were not returned by the search.

### 2.2. Micronutrients

Micronutrients are categorized as having four main roles in supporting major body function: cofactors in metabolism, coenzymes in metabolism, genetic controls, and antioxidants [15]. Therefore, deficiency of essential micronutrients leads to deleterious downstream effects involving metabolism, gene expression and oxidative stress [15]. In the context of IBD, the role of micronutrients in genetic control and antioxidation are of particular importance [16]. Vitamin A, B, and E have been implicated in regulating immune responses and homeostasis within the gut, inhibiting the release of inflammatory cytokines and favoring the differentiation of regulatory (T_REG_) lymphocytes over proinflammatory T_H_17 lymphocytes [16]. In healthy patients, production of reactive oxygen species (ROS) is balanced by the activity of antioxidative micronutrients such as vitamins A and E, as well as enzymes requiring trace metals (zinc and selenium as examples) as cofactors [16]. In IBD, immune dysregulation and inflammation result in the increased production of ROS, further underlying the importance of antioxidative micronutrients in mitigating IBD pathogenesis [17]. This relationship creates a chicken and the egg situation: Is increasing disease severity responsible for micronutrient deficiencies by impairing absorption? Or are dwindling levels of micronutrients, which act as antioxidants and immune regulators, responsible for progression of disease?

### 2.3. Vitamins

#### 2.3.1. Vitamin A

Vitamin A, and its active metabolite retinoic acid, have widespread effects through the body. In the context of the immune response, retinoic acid signaling results in expression of Foxp3, a transcription factor that leads to the differentiation of naïve T cells to T_REG_ cells [18,19,20,21]. These T_REG_ cells attenuate the immune response through the release of anti-inflammatory cytokines [22,23]. Conversely, retinoic acid inhibits the expression of IL-6 receptors, preventing the differentiation of naïve cells into proinflammatory T_H_17 cells [20]. Furthermore, retinoic acid induces the expression of α4β7 and CCR9 on T cells [24]. The expression of these molecules causes T cells to preferentially migrate into the gut wall following exposure to antigen [25]. 

Vitamin A absorption chiefly occurs in the proximal intestine and can be directly converted into retinoic acid in the gut by enterocytes [26]. Given that vitamin A stores in the liver buffer decreasing levels in the blood, measuring serum vitamin A does not accurately assess the total amount present in the body [27]. Soares-Mota et al., 2015 used relative dose response testing to indirectly assess liver stores of vitamin A in 38 Brazilian patients with CD and 34 healthy subjects. They found that deficiencies in vitamin A stores were significantly more common in those suffering from CD (37% vs. 12%, *p* < 0.005). Interestingly, they did not find any association between vitamin A deficiency, disease location, or disease activity [27]. 

Provitamins of vitamin A have also been examined in the context of IBD. One such provitamin, β-carotene, has been well studied [28]. Compared to controls, β-carotene levels in IBD patients have been shown to be significantly decreased, with further decrease in those with active disease [29]. As well, supplementation with β-carotene lead to disease amelioration in a dextran sodium sulfate (DSS) murine model of colitis [30]. Conclusions are much harder to draw from results of human trials. Firstly, in the articles reviewed by Rezaie et al., 2007, each study uses β-carotene alongside other antioxidative micronutrients, making it difficult to judge its effect [31]. Furthermore, the review notes that the studies used small doses of β-carotene and did not see normalization of β-carotene serum levels. The one study reviewed that used high dose antioxidants, including β-carotene, saw decreases in the required corticosteroid dose, while all other studies were negative [31,32]. Again, without elucidating the effect of β-carotene supplementation in isolation, it is impossible to determine its clinical utility. It is also difficult to distinguish whether any effects are due to its conversion to vitamin A or its own antioxidative ability.

The role of non-provitamin A carotenoids in IBD will only be described briefly, as their role in the context of IBD requires further elucidation. Presently, deficiencies of these carotenoids, namely lycopene and lutein, have been described in IBD patients [30,33]. However, the clinical significance of these deficiencies in IBD is unclear. While the effect of dietary supplementation has not been determined, a single retrospective study using the self-reported dietary records of 56 UC patients in remission associated higher lycopene and lutein intake with decreased fecal blood and mucus, with no change in abdominal pain [34]. Lutein was also able to attenuate ulceration of the GI tract in DSS murine models [35]. Given the exceeding rarity of clinical serum testing for these compounds, and the lack of data surrounding their supplementation, significant further research is required before any recommendation can be made.

Given the ability of vitamin A to attenuate intestinal inflammation in murine models and the prevalence of deficiency in the Brazilian CD population, further research into the role of vitamin A in IBD is warranted [27,36]. At this point, research regarding vitamin A supplementation in IBD is limited to two small studies published in 1985, (Wright et al., 1985: *n* = 86; Norrby et al., 1985, *n* = 8) [37,38]. Neither of these papers found a significant benefit of supplementation. However, drastic changes in the management and treatment of IBD over the past 30 years, combined with small sample sizes in both studies make this an attractive topic to revisit using well-designed clinical trials [39].

#### 2.3.2. Folate

While folate (also known as vitamin B9) deficiency has classically been associated with anemia in IBD patients, it’s role in IBD is likely more complex than previously thought. Not only is folate essential in the synthesis of nucleotides, but it is implicated in the regulation of T_REG_ cells [40]. In vitro, folate acts as a pro-survival signal for T_REG_ cells [41,42]. Similarly, in murine models, deficiency in folate leads to the reduction of T_REG_ cells in the intestine [40]. Murine models have also demonstrated a predisposition towards intestinal inflammation in mice being fed diets deficient in folate [42,43]. Furthermore, folate deficiency (as well as B12 deficiency) inhibits the conversion of homocysteine to methionine, which results in increased levels of homocysteine in the blood and intestinal mucosa of a significant portion of IBD patients [44]. Homocysteine acts as a pro-oxidant amino acid, increasing oxidative stress in the body [45]. Homocysteine also promotes the secretion of pro-inflammatory chemokines, and the differentiation of naïve CD4+ T cells into pro-inflammatory T_H_17 cells [46]. Homocysteine levels are correlated with thrombotic events, and interestingly, IBD patients have an increased prevalence of thrombosis compared to those without IBD (5-year prevalence, 7.5% vs. 4.5%, *p* < 0.001) [47]. Furthermore, the patients are at increased risk of mesenteric ischemia (HR 11.2), and female IBD patients have significantly higher risk of MI (HR 1.6) and stroke (HR 2.1) [48].

Folate is absorbed in the jejunum following its conversion from folate polyglutamate to monoglutamate in the gut [49]. Further conversion in the liver results in the production of the metabolically active tetrahydrofolate (THF) [49]. Folate levels are significantly lower in patients with both CD and UC, with one prospective study of 180 IBD patients suggesting that 22.2% of CD patients and 4.3% of UC patients are deficient [50]. While the mechanism behind folate deficiency has not been well categorized, jejunal dysfunction and reduced intake of folate-containing foods are likely contributors. Furthermore, the use of methotrexate or sulfasalazine in IBD treatment leads to errors of folate metabolism and absorption [51,52].

While anemia is common in IBD patients, the authors were unable to find a study that assessed the proportion of patients with megaloblastic anemias in the setting of folate deficiency. Furthermore, folate supplementation remains a relatively unexplored topic. At this point, only the effect of supplementation on colorectal cancer has been described. A meta-analysis of 10 such articles determined that folate supplementation significantly lowers the risk of colorectal cancer in IBD patients (HR 0.58; 95% CI: 0.36–0.66) [53]. However, the studies analyzed used varying doses of folate, making recommendations difficult. The effect of folate supplementation on homocysteine levels in the intestinal mucosa, and the risk of thrombotic event, are logical targets of future research.

#### 2.3.3. Vitamin B12

Unlike the micronutrients discussed above, no direct connection between vitamin B12 (cobalamin) and immune regulation has been described. Vitamin B12 has been included in this review due to the clinical consequences of its deficiency, as well as the impact of CD on the site of its intestinal absorption. Vitamin B12 is essential in the metabolism of macronutrients, synthesis of DNA, and nerve function [54]. Deficiency is associated with macrocytic anemia via impaired folate metabolism, neurologic damage, and hyperhomocysteinemia [54]. While serious, these complications are uncommonly reported in IBD patients. In order to be absorbed, vitamin B12 requires activation by intrinsic factor produced in the stomach. Following activation, vitamin B12 is only absorbed via specific receptors present in the terminal ileum [55]. This location makes its deficiency a particular concern in CD, as a significant proportion of CD patients develop ileal disease [56].

Numerous studies exist that assess B12 status in IBD patients and the results vary [50,57,58,59,60]. In recent years, both a systemic review and meta-analysis of the literature have concluded that IBD is not associated with significantly lower serum B12 levels [61]. However, surgical resection of the ileum is associated with B12 deficiency [61]. Meta-analysis by Pan et al., 2017 suggests that patients with ileal resections were disproportionately underrepresented in the literature surrounding B12 deficiency [61]. Considering that ileal disease is common in CD, and upwards of 50% of CD patients require surgery within 10 years of diagnosis, patients with ileal resection, and therefore B12 deficiency, likely represent a larger group than described [62].

#### 2.3.4. Vitamin D

The relationship of IBD and vitamin D is likely the most complicated of the IBD-micronutrient relationships. While normally associated with calcium metabolism and bone health, the role of vitamin D in the regulation of the immune response has been the topic of much research in recent years [16]. In the gut, vitamin D’s active form 1α,25-dihydroxyvitamin D3 (calcitriol) alters the T cell response, favoring T_REG_ cells and inhibiting production of proinflammatory cytokines [63]. Calcitriol also impairs the function of interstitial dendritic cells, limiting their ability to activate T cells within the gut [63]. Furthermore, direct signaling via vitamin D receptors on T cells leads to upregulation of programmed death receptors and the downregulation of the activating receptor CD69 [64]. These properties provide a rational physiological means by which vitamin D deficiency affects IBD pathogenesis. This is further substantiated by murine models of IBD in which vitamin D supplementation improves symptoms, and deficiency increases symptoms [65,66].

Vitamin D is acquired through the diet, however the majority of vitamin D is synthesized endogenously in the skin [67]. Once acquired, vitamin D must then be metabolized step-wise in the liver and kidney into its active form 1α,25-dihydroxyvitamin D3 [67]. Vitamin D deficiency is common in IBD patients, with a meta-analysis identifying deficient vitamin D levels in 57.7% of CD patients [68]. The exact reason for vitamin D deficiency in IBD is not well understood. Considering that few foods naturally contain vitamin D, deficiency is likely a combination of reduced intake to diet restrictions and aversions, as well as impairment of the absorption of fat-soluble vitamins [69].

Literature correlating vitamin D deficiency with IBD disease activity is abundant and relatively homogenous [70,71,72,73,74,75]. However, there exists a limited amount of data in patients whose vitamin D levels have been restored through supplementation. Broadly, vitamin D levels are inversely associated with inflammatory markers, while positively associated with bone mineral density (BMD), anti-inflammatory cytokine production, and response to anti-tumor necrosis factor (TNF) therapy [70,76,77,78,79,80]. In both CD and UC, vitamin D deficiency is also associated with more frequent relapses of disease, disease severity, risk of colorectal cancer [81,82,83]. Similarly, a landmark longitudinal study of 965 American IBD patients saw a significant association between low vitamin D concentration and increased utilization of healthcare, increased pain, and decreased quality of life [84]. This study also found that vitamin D normalization significantly reduced healthcare utilization in the same population [84]. Ananthakrishnan et al., 2013 have corroborated this finding, showing that normalization of vitamin D levels reduces risk of surgery in CD patients [85]. In terms of BMD, concurrent supplementation with calcium and vitamin D led to significantly increased BMD in the hip and lumbar spine [86,87].

Dosing of vitamin D for effect in IBD is not well-established [88]. Doses of vitamin D3 at 4000 IU/day are adequate in correcting deficiency but given the conflicting evidence around the effect of supplementation on disease course in the first place, the most appropriate therapeutic dose has not been determined [88,89].

#### 2.3.5. Calcium

Calcium homeostasis is a complex, multifaceted phenomenon. Bone, which is 60% comprised of calcium hydroxyapatite (Ca_10_(PO_4_)_6_(OH)_2_, undergoes constant remodeling throughout life. Therefore, the pathways responsible for the control of calcium homeostasis are critically important. The supply of calcium involves paracellular and transcellular intestinal and renal absorption and reabsorption [90]. Chronic intestinal inflammation of IBD, as well as dietary and lifestyle alterations and medications used to treat IBD, impact these physiologic homeostatic pathways [7,9,87,90,91]. Intestinal malabsorption, as a result of decreased dairy product intake, leads to low luminal ionic calcium (Ca^2+^) concentrations resulting in increased transcellular active transport (versus more non-active paracellular transport when luminal Ca^2+^ concentrations are high via decreased duodenal transit time and down regulation of protein responsible for transcellular Ca^2+^ transport proteins). Ca^2+^ is absorbed through facilitated diffusion mediated by transient receptor potential villanoid channels (TRPV6 and 5), which are regulated by vitamin D3, parathyroid hormone, estrogens, pH- and Ca^2+^ dependent regulatory mechanisms, and bidirectional Na+/Ca^2+^ exchange [90].

Beyond the known negative influence of vitamin D insufficiency and malnutrition, there is a relative paucity of knowledge relating to the impact of acute and chronic intestinal inflammation itself on Ca^2+^ homeostasis in the kidneys and intestine. A study by Huybers et al. demonstrated in a TNF-α overexpressing mouse model of Crohn’s ileitis that multiple epithelial proteins involved in Ca^2+^ absorption and transport, including TRPV6, were down regulated [92]. As a result of changes in the expression of Ca^2+^ transport genes, bone resorption increased. When these same mice were treated with methylprednisone, similar inhibitory effects were seen in TRPV6 and calbindin D9K mRNA and protein. Although limited data exists in IBD, a very small (*n* = 4) study of CD patients in remission revealed intestinal absorption of Ca^2+^, as well as response to 1,25(OH)_2_D_3_, to be similar to that of controls [92].

In addition to calcium, phosphate (P_i_) homeostasis is important in the maintenance of BMD. Prolonged phosphate deficiency may lead to bone demineralization (osteomalacia and rickets). Although little is known about the impact of intestinal inflammation on P_i_ absorption, as with serum Ca^2+^ it is known that serum levels of P_i_ do not reflect minor alterations in active mineral transport. Therefore, it is very uncommon to observe changes in serum Ca^2+^ or P_i_ in IBD patients. The *Klotho* gene, a multifunctional protein, has been implicated in the regulation of both Ca^2+^ and P_i_ metabolism through its influence on transepithelial Ca^2+^ transport via promotion of transport channel recruitment and retention. Thurston et al., have demonstrated renal Klotho to be significantly down-regulated in various murine models of colitis via TNF and IFN-gamma [93]. 

Through complex absorptive, transport, and hydroxylation mechanisms, vitamin D3 is converted to the hormonally active form of vitamin D, 1,25(OH)_2_D_3_, which is responsible for most, if not all, of the biological effects of vitamin D. 1,25(OH)_2_D_3_ has a pleiotropic role and is a key regulator of bone turn-over, with the potential to facilitate both osteoclastic and osteoblastic pathways. Numerous studies have demonstrated the association between vitamin D deficiency or insufficiency and IBD when compared with control or healthy individuals [94,95]. The mechanisms for this observed associations in IBD may include intestinal malabsorption, decreased dietary intake, disrupted enterohepatic circulation, renal insufficiency and inadequate sunshine exposure. Given the association between IBD and decreased vitamin D status, numerous interventional studies have been conducted in an attempt to determine the impact of vitamin D supplementation on IBD-related clinical outcomes. The data related to BMD loss or accrual have been mixed [90,96,97]. In fact, there have been several studies that suggest, in the setting of active inflammation, that high concentrations of 1,25(OH)_2_D_3_ may promote the development of osteoporosis in CD [98]. Likewise, the outcomes of studies relating to bone formation and resorption in IBD patients have been conflicting, likely due to flaws in study design, power, confounding factors, IBD complexity, and challenges related to understanding and measuring bone turn-over rates in both adults and children with IBD. More well designed and adequately powered clinical studies are required to definitely answer these questions relating to bone formation and resorption in IBD [90].

The presumption that the BMD effects observed in IBD are simply as a result of vitamin D3 and calcium deficiency has more recently been recognized to be overly simplistic. The complex interplay between the inflammatory disease process itself and the mediators of mineral homeostasis and bone (re)absorption complicates efforts to clearly elucidate the role that these nutrients and hormones play in the pathogenesis of metabolic bone disease in patients with IBD, as well as the role that acute and chronic inflammation may play in alterations of mineral and hormonal homeostasis.

#### 2.3.6. Vitamin K

The mechanism of vitamin K absorption is two-fold. Vitamin K is both directly absorbed from the diet and produced in the gut by commensal GI flora. As a cofactor, vitamin K is crucial to the production of coagulation factors and in the maintenance of bone [99]. As well, much like many of the other micronutrients discussed, vitamin K is implicated in immune signaling and inflammation [16]. Poor vitamin K levels are associated with increasing levels of IL-6 in the blood, a pro-inflammatory cytokine that is implicated in IBD pathology [100,101]. Similarly, in murine models, vitamin K supplementation reduced intestinal inflammation in the presence of the bacterial antigen lipopolysaccharide (LPS), while deficiency aggravated disease in DSS models of colitis in mice [102,103]. This provides a dual rationale for vitamin K supplementation in IBD: avoid complications such as bleeding and bone loss, while hopefully pushing the body away from pro-inflammatory immune pathways.

Vitamin K deficiency is found in 31% of IBD patients, with dysregulation of the gut biota and bile salt malabsorption proposed as mechanisms [69]. In these patients, low levels of vitamin K are associated with low BMD, as well as clinical activity and duration of disease [104,105,106]. In long-standing IBD, the prevalence of osteoporosis can be as high as 41% [107]. Considering this, and the adverse outcomes associated with fractures, BMD is of critical importance in IBD management. Daily supplementation of vitamin K over 12 months was unable to alter the indices of bone health in IBD patients with vitamin K deficiencies [108]. Furthermore, in rheumatoid arthritis, which shares some common inflammatory pathophysiology with IBD, vitamin K supplementation did not significantly alter the level of IL-6 or the clinical activity of disease in patients [109]. Given the link of vitamin K deficiency to low BMD and high IL-6, these results are disappointing.

### 2.4. Metals

#### 2.4.1. Iron

Aside from vitamin D, iron is likely the micronutrient that has been most heavily studied in the context of IBD. Iron has attracted attention due to the high portion of IBD patients who suffer from anemia [110]. The mean prevalence of anemia calculated by meta-analysis is 16% in outpatients, increasing to 68% in inpatients [99]. As well, the overall portion of IBD patients with iron deficiency is 45% [111]. The physiological means by which IBD causes iron-deficiency is straight forward. Mucosal bleeding leads to iron loss in the stool, meanwhile inflammatory changes altering hepcidin and iron metabolism impact iron absorption [112].

Anemia, and the associated fatigue, can be one of the most crippling symptoms of IBD, with quality of life scores mirroring hemoglobin (Hb) levels [113]. On this basis alone, the treatment of iron-deficiency anemia in IBD is paramount. Current recommendations suggest the end goal of iron supplementation is the normalization of iron store levels, as well as Hb [114]. Oral and intravenous iron products are available for use in IBD. Clinically, and in meta-analysis, intravenous iron supplementation is associated with significantly fewer GI side effects, as well as slightly greater increases in Hb [115]. However, the patient’s quality of life did not significantly differ between intravenous and oral iron [116]. Importantly, anemia reoccurs rapidly in IBD, further necessitating the use of iron maintenance therapies [117]. In line with this, European Crohn’s and Colitis Organization (ECCO) guidelines suggest that patients who have been treated for iron-deficiency anemia be monitored every 3 months for the first year, and every 6–12 months for the recurrence of anemia [114].

#### 2.4.2. Zinc

As a cofactor, zinc is intimately involved in the regulation of both the innate and adaptive arms of the immune system [118]. Importantly, zinc downregulates NF-κB signaling through the zinc-finger protein A20, leading to decreased expression of its downstream products (namely IL-6 and TNF-α) [119,120,121]. It is worth noting that certain mutations in the gene that encodes A20 (*TNFAIP3*) are positively associated with the risk of developing numerous inflammatory conditions, including IBD [121]. As well, deletion of *TNFAIP3* in mice intestinal cells leads to sensitivity to DSS-induced colitis. Conversely, zinc deficiency results in increased production of pro-inflammatory molecules in murine models [122]. Finally, zinc also exerts an anti-oxidative effect through its inhibition of NADPH oxidase, and its role as a cofactor for superoxide dismutase [123,124]. The relationship between zinc and IBD on both a theoretical and genetic level, make it a micronutrient of interest to explore further.

Zinc deficiency is present in a significant portion of IBD patients (15.2–65%) [8,125]. Zinc levels are balanced through absorption in the proximal small intestine, and excretion in intestinal and pancreatic secretions [126]. Losses are increased in association with chronic diarrhea, high output ostomies, and high output fistulas, which often pose a problem for IBD patients. These increased losses combined with the malabsorptive state of intestinal inflammation are likely responsible for zinc deficiency in IBD patients.

Clinically, an association has also been observed between zinc deficiency and IBD. A prospective study of 170,756 women over 26 years found that increasing zinc intake was inversely corelated with CD risk [127]. Furthermore, an American cohort study of 773 CD and 233 UC patients highlighted the relationship between zinc deficiency and adverse patient outcomes. They found that zinc deficiency was associated with a significant increase in hospitalizations (CD: OR 1.44. 95% CI [1.02–2.04]; UC: OR 2.14, 95% CI [1.07–4.29]), surgery (CD: OR 2.05, 95% CI [1.38–3.05]; UC: OR 1.64, 95% CI [0.59–4.52]), and complications (CD: OR 1.50, 95% CI [1.04–2.15]; UC: OR 1.97, 95% CI [0.94–4.11]) [128]. In patients who were able to achieve normal zinc levels within 12 months, the risk of adverse outcomes returned to baseline [128]. Although zinc deficiency is associated with disease activity and worse outcomes, a single small cross-over trial (*n* = 14) that explored the effect of daily zinc supplementation did not show changes in the level of pro-inflammatory cytokines, or disease activity [129]. Given the positive results seen by Siva et al., 2017, larger trials following zinc supplementation may be a reasonable step forward [128].

#### 2.4.3. Selenium

Much like zinc, selenium is integral to the immune function. Selenoproteins, through which selenium exerts its effect, encompass a group of proteins with diverse pro- and anti-inflammatory effects that are beyond the scope of this review. Relevant to IBD, glutathione peroxidases (GPx) utilize selenium to mitigate the effect of ROS [130]. Whereas loss of the genes encoding GPx proteins results in increased severity of disease in DSS murine models [131,132,133].

Selenium deficiency is common in IBD patients with a recent study reporting 30.9% of patients as deficient, and numerous others observing levels that were significantly decreased compared to control [134,135,136,137,138,139]. In DSS-treated mice, selenium deficiency leads to increased severity of disease and upregulation of pro-inflammatory TH1 cytokines [140,141]. In humans, selenium deficiency has been investigated for its possible role in cardiovascular disease, however a recent review by Benstoem et al., 2015 describes the literature as “inconclusive” [142].

Selenium supplementation in the context of IBD has not been documented in the literature outside of murine models. In these models, supplementation ameliorated cox-dependent inflammation by altering the PGE2 pathway [143,144]. Given the abundance of animal data, and the strong physiological link between selenium and immune function, the study of selenium therapy should be a future focus. Table 1 presents the immune implications of micronutrient deficiencies, including selenium, in adult IBD patients.

## 3. Recommendations and Conclusions

The understanding of the role of micronutrients in immune system function has changed greatly in recent years, as has our understanding of the pathophysiologic mechanism(s) of Inflammatory Bowel Disease (IBD). However, the etiology of IBD and the role that micronutrients play in the development of IBD is still unclear. There are clear physiologic links between immune-regulating micronutrients and the balance of pro- and anti-inflammatory processes in the gut (see Table 1) [16]. However, experimental models often oversimplify what is experienced clinically (see Table 2). The same is true of micronutrients with anti-oxidative effects [16,17]. In addition, the bidirectional relationship of deficiency and disease makes the exact role of each micronutrient difficult to characterize, as IBD impacts nutritional status through both decreased intake (impaired absorption, avoidance of trigger foods, decreased appetite, decreased surface area secondary to resection, etc.), and increased gastric losses (chronic diarrhea, intestinal blood loss, mucus losses, etc.). The effect of micronutrients on outcomes is most easily understood in the context of iron, in which IBD leads to iron deficiency, and deficiency leads to anemia. However, for the other micronutrients, the relationship between IBD, deficiency, and clinical outcomes is much less clear. Table 2 presents the clinical outcomes associated with level of micronutrients discussed in this paper, in adult IBD patients.

hile association studies suggest the benefit of treating deficiency of iron, zinc and vitamin D, similar studies have not been performed for other micronutrients. Similarly, literature regarding the use of supplementation as therapy is limited to a few, small scale studies that only targeted specific subgroups of patients [37,38,129]. These factors make specific recommendations on dosage for supplementation difficult. At this point, current European Society for Clinical Nutrition and Metabolism (ESPEN) recommendations suggest that micronutrient levels be assessed annually in IBD patients, with the correction of any deficit with supplementation [145]. Guidelines for iron supplementation are more specific, suggesting iron repletion therapy in all IBD patients with iron-deficiency anemia, with the end goal of therapy being the normalization of both hemoglobin and iron stores [114]. Intravenous iron should be considered in patients with clinically active disease and those who are intolerant of oral iron, while oral iron should be considered in those with inactive disease or milder anemia [114]. The dose of iron is dependent on the route and formulation chosen, however a response of 2 g/dL within 4 weeks is appropriate [114]. As for zinc, a 3-month course of daily supplementation with 200 mg of ZnSO_4_ normalizes zinc serum concentrations in deficient IBD patients [146]. Given that zinc citrate has similar bioavailability and is more palatable to patients its use may be preferred [147]. In addition, one author suggests increasing zinc supplementation in patients with diarrhea in order to offset increased GI loses, at a rate of 30–40 mg elemental zinc for every liter of stool output [148].

For the other nutrients discussed in this paper, evidence-based recommendations for supplementation in the context of IBD do not exist. Rather, clinicians should focus on the correction of any micronutrient deficiencies that are found using appropriate oral therapy. Previous authors have posited varying suggestions on dosage with regards to reversing deficit, with high dose oral therapy as the common theme among them. A 2003 review by Eiden highlights such suggestions in IBD patients and is a valuable reference for clinicians with deficient IBD patients [149].

The development of specific recommendations for supplementation and micronutrient levels to targets is a priority. In order to do so, large, well-designed, controlled studies focused on supplementation are required. Given the consistency of the observed association between zinc and vitamin D and IBD clinical disease activity and inflammation, further study of supplementation of vitamin D and zinc as an adjunct to therapy is appropriate [16,36]. Given their link to immunity and oxidation, further characterization of the role of vitamin A, B9, and K in IBD pathogenesis is also warranted.

## Figures and Tables

**Table 1:** Immune implications of micronutrient deficiencies in IBD.

Micronutrient	Level in IBD	Purported Pathogenic Role of Deficiency
Vitamin A	↓ [27]	↓ T_REG_ cell differentiation [18,19,20,21] ↑ T_H17_ cell differentiation [20] ↓ T cell migration into the gut [25]
Folate	↓ [43]	↓ T_REG_ cell survival [31,32] ↑ Proinflammatory signaling [37,39] ↑ Oxidative stress [37,38]
Vitamin B12	=↓ in patients with ileal resection [54]	--
Vitamin D	↓ [61]	↑ T cell activation [56] ↑ Proinflammatory signaling [56] ↓ Decreased T cell turnover [57]
Calcium	↓ [87,90,91]	--
Vitamin K	↓ [62]	↑ Proinflammatory signaling [84,85]
Iron	↓ [104]	--
Zinc	↓ [8,118]	↑ Proinflammatory signaling [114] ↑ Oxidative stress [116,117]
Selenium	↓ [134,135,136,137,138,139]	↑ Proinflammatory signaling [141] ↑ Oxidative stress [130]

This table presents the immune implications of micronutrient deficiencies in adult IBD patients. The arrows describe the effect of IBD on the level of micronutrient present in the individual, as well as the effect on the purported pathogenic role related to that micronutrient. For example, ↑ indicates an increase, ↓ indicates a decrease, and =↓indicates about the same or less.

**Table 2:** Clinical outcomes associated with micronutrient levels in IBD.

Micronutrient	Effect of Deficiency	Effect of Supplementation
Vitamin A	--	--
Folate	Macrocytic anemia ↑ Homocysteinemia [44]	↓ Risk of colorectal cancer [53]
Vitamin B12	Macrocytic anemia ↑ Homocysteinemia Neurologic damage [54]	--
Vitamin D	↑ Inflammatory markers [70,77] ↑ Healthcare utilization [84] ↑ Disease activity [70,71,72,73,74,75] ↑ Risk of colorectal cancer [83] ↓Quality of life [72,85]	↑ Bone mineral density ↓ Risk of surgery
Vitamin K	↓ Bone mineral density [104,105,106] ↑ Bleeding risk [99]	↔ Disease activity ↔ Bone mineral density
Iron	Iron-deficiency anemia	Resolution of anemia ↑ Quality of life [113]
Zinc	↑ CD risk [127] ↑ Hospitalizations ↑ Surgery ↑ Complications [126]	↔Disease activity
Selenium	? Cardiovascular disease [142]	--

This table presents the clinical outcomes associated with micronutrient levels in IBD. The arrows describe the effect of IBD on the level of micronutrient present in the individual, as well as the effect on the purported pathogenic role related to that micronutrient. For example, ↑ indicates an increase, ↓ indicates a decrease, ↔ indicates a potential increase or decrease, and ? indicates that the outcome is unknown.
